# Knee Implant Loosening Detection: A Vibration Analysis Investigation

**DOI:** 10.1007/s10439-017-1941-2

**Published:** 2017-10-24

**Authors:** Arash Arami, Jean-Romain Delaloye, Hossein Rouhani, Brigitte M. Jolles, Kamiar Aminian

**Affiliations:** 1 Laboratory of Movement Analysis and Measurement, Ecole Polytechnique Fédérale de Lausanne (EPFL), Lausanne, Switzerland; 2 Centre Hospitalier Universitaire Vaudois (CHUV), Lausanne, Switzerland; 3 Human Robotics Group, Department of Bioengineering, Imperial College London, London, UK; 4 Department of Mechanical Engineering, University of Alberta, Edmonton, Canada; 5 University of Lausanne (UNIL), Lausanne, Switzerland

**Keywords:** Knee prosthesis, Implant loosening, Vibration analysis, Power spectrum density, Input–output coherence

## Abstract

Knee implant loosening is mainly caused by the weakness of the prosthesis-bone interface and is the main reason for surgical revisions. However, pre-operative diagnosis is difficult due to lack of accurate tests. In this study, we developed a vibration-based system to detect the loosening of the tibial implant of an instrumented knee prosthesis. The proposed system includes an instrumented vibrator for transcutaneous stimulation of the bone in a repeatable manner, and accelerometer sensors integrated into the implants to measure the propagated vibration. A coherence-based detection technique was proposed to distinguish the loosened implants from the secure ones. Fourteen *ex vivo* lower limbs were used, on which the knee prosthesis was implanted, and harmonic-forced vibration was applied on the tibia. The input–output coherence measure provided 92.26% accuracy, a high sensitivity (91.67%) and specificity (92.86%). This technique was benchmarked against power spectrum based analysis of the propagated vibration to the implant. In particular, loosening detection based on new peak appearance, peak shift, and peak flattening in power spectra showed inferior performance to the proposed coherence-based technique. As such, application of vibration on our instrumented knee prosthesis together with input–output coherence analysis enabled us to distinguish the secure from loose implants.

## Introduction

Osteoarthritis is the most common joint disease, the primary cause of joint pain and disability in the elderly population. The knee is the main articulation involved and the risk of developing symptomatic knee osteoarthritis during a person’s lifetime has been estimated over 40%.^28^ Total knee arthroplasty (TKA) is a successful and widely performed procedure for end-stage osteoarthritis. TKA operations, which have been dramatically increased, recently outnumbered the performed hip replacements.^8^ This increment precipitated an increasing need for TKA revisions, especially in younger and active patients.^8^ TKA revisions could increase by six-fold from 2005 to 2030.^21^ Aseptic implant loosening remains the main cause for this procedure associated with more than 30% of all TKA revisions, and tibial implant being mostly involved.^8^ Nonetheless, diagnosis of aseptic TKA loosening is still a challenge.

X-ray remains an informative, quick, and inexpensive method to diagnose implant loosening. However, the described Knee Society’s criteria for TKA loosening^13^ depend on the exam quality,^7^,^29^ and showed a poor accuracy suggesting that X-ray cannot reliably detect early prosthesis debonding.^38^,^43^ Recently, techniques such as radionuclide arthrography,^20^ 18-fluorodeoxyglucose positron emission tomography,^12^,^43^ single photon emission computerized tomography associated with CT-scan, or bone scintigraphy to detect implant loosening have been studied.^1^,^9^,^42^ However, their TKA loosening detection performances are variable. Bone scintigraphy is widely used, despite the inherent difficulty in image interpretation,^25^,^37^ being time-consuming and costly, and reported unreliable in detecting implant loosening.^31^

The vibration and modal analysis, which has been broadly used for structural integrity and damage analysis,^18^,^39^ is another class of methods for prosthesis loosening detection. After a successful implantation, the implant and bone form unit system, while any crack or mechanical stiffness deviation can result in natural frequencies alternations.

Early studies in total hip arthroplasty (THA) loosening focused on the transmission of sinusoidal vibration waves to the implants,^24^,^37^ where the non-sinusoidal output waveform indicated THA loosening. Other researchers used a simple hand-held set-up to vibrate and measure acceleration over the bony landmarks of the tibia and femur and studied the frequency domain amplitude response of the system.^16^ However, their set-up suffered from inaccuracies due to the change in hand-held contacts and the soft tissue attenuation effect. Although integrating sensors in the ball head of the THA can reduce the inaccuracies,^33^ the inaccuracies related to the shaker contact still remained. A complete wireless measurement system with integrated sensors and electronics was introduced and tested on the bare femur and artificial thigh.^26^ Using finite-element models, Qi *et al*.^34^ investigated the vibration analysis for THA loosening and showed that the peak shifts and harmonics can appear in different frequency bands and could be indicators of implant loosening with different sensitivities. Several vibration analysis techniques including the linearity of system response to Gaussian input were simulated.^44^ A device was designed to excite the bone-implant system and estimate the frequency response function before, during and after application of an external torque to analyze the THA stability.^23^,^46^ Vibration analysis was performed for preoperative monitoring of the THA integration in which the evolution of frequency response function at various cement curing stages was demonstrated.^32^ In another study,^35^ six sawbone models were used for THA loosening detection with a discrimination between the cup and stem loosening. The number of peaks in spectra was used to detect the cup loosening.

To design a reliable system for TKA loosening detection, the general methodology of previous studies can be used. To compare the bone-prosthesis system responses before and after implant loosening the sensor and vibrator placements and the boundary conditions must be accurately controlled and repeated. To increase the sensitivity of TKA loosening detection, the sensors can be integrated into the prosthesis itself. However, current studies on instrumented prostheses mainly focused on measurements of force, moments and kinematics,^2^,^4^,^5^,^11^,^19^,^22^ and none were designed for *in vivo* implant loosening detection.

In this study, we targeted the design and feasibility study of a vibration-based system to detect cemented knee tibial implant loosening in an objective, facile and repeatable way. Since the geometry and bone-implant contact in TKA are totally different from THA, new and potentially different features need to be extracted and investigated. We hypothesized that tibial implant loosening can be detected based on the alternation of the frequency response of the tibia-cement-implant to repeatable vibrational stimuli and the identified discriminative features are repeatable among a population. Particularly we hypothesized that input–output vibration coherence can present a discriminative feature for loosening detection.

## Materials and Methods

### Vibration System and Measurement Units

**Figure 1:**
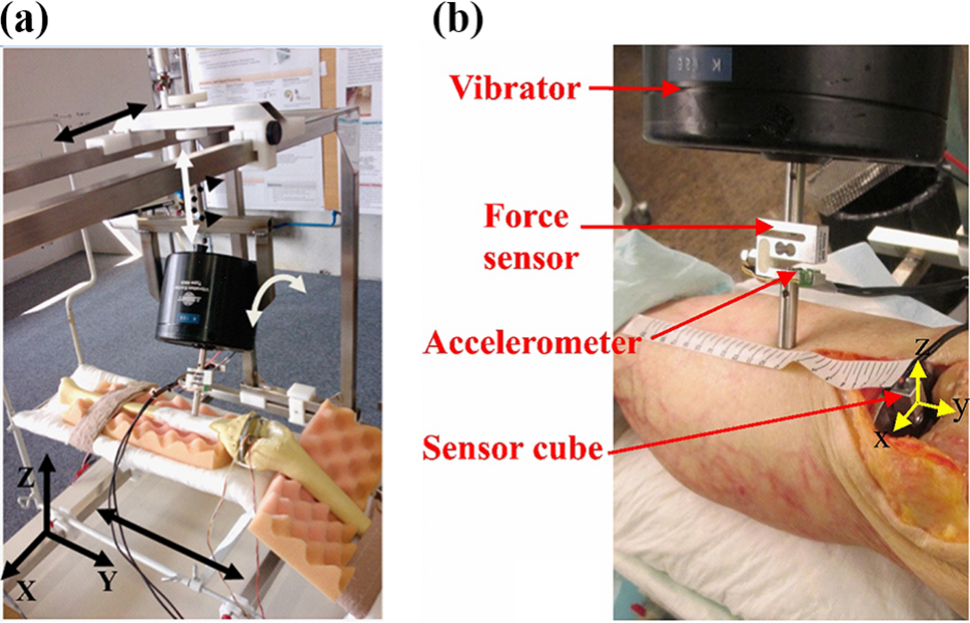
(**a**) Vibrator oriented in the adjustable apparatus over a saw bone, (**b**) a closer view of the stimulation system and the measurement systems during a cadaveric experiment.

A vibrator system (Exciter type 4809, Brüel & Kjær, Denmark) was used to provide the input vibration (stimulus) to the bone through skin interface. The actuator tip was not bolted but was firmly in contact with skin and applied oscillating compressive force (Fig. 1). Input vibration was controlled using an operational power amplifier (BOP72-6 M, KEPCO, USA) and a signal generator (HP3314a, Hewlett-Packard, USA). Stimuli had a sinusoidal waveform, twice linearly swept from 30 Hz to 3 kHz. The resultant chirp signal provides rich stimuli which have been recommended and widely used for system identification and modal analysis problems.^30^,^40^

To generate repeatable stimuli for the bone-cement-implant system, we built an apparatus (Fig. 1a) which provides 3 degrees of freedom (DOF) for displacement (*X*, *Y*, and *Z* axes) and 2 DOF for rotation (around *Y* and *Z* axes) for the vibrator. This apparatus was securely fixed to the surgical table. The leg was fixed on a knee implantation bed, fixed on the apparatus (Fig. 1a), to provide an approximate flexion angle of 45°. This apparatus facilitated the manipulation and fixation of the vibrator to impact on anatomical positions with desired impact angle, avoided the slipping of exciter tip over the skin and allowed repeatable stimulation.

A 2D accelerometer [ADXL203 family, Analog Devices, USA, range: ± 5 g, min bandwidth: (0.5 Hz 5.5 kHz)], selected to match the vibration amplitude and frequency ranges of the stimuli, was fixed on the vibrator tip (Fig. 1b). It measured the axial and lateral vibrations and was also used as an inclinometer, before the test, to control the vibrator tip impact angle. A force sensor (KD40S, ME-measurement, Germany) was sandwiched in the vibrator pole to measure the applied force to the bone before and during the vibrations (Fig. 1b). The force was monitored in real-time before starting the stimulation, allowed us to control the vibrator-bone contact and maintain an identical contact force across different experiments. All sensors were linked to a data acquisition board (NI-DAQ6016, National Instrument, USA) which acquired data with 12-bit ADC resolution and at a sampling frequency of 8 kHz.

**Figure 2:**
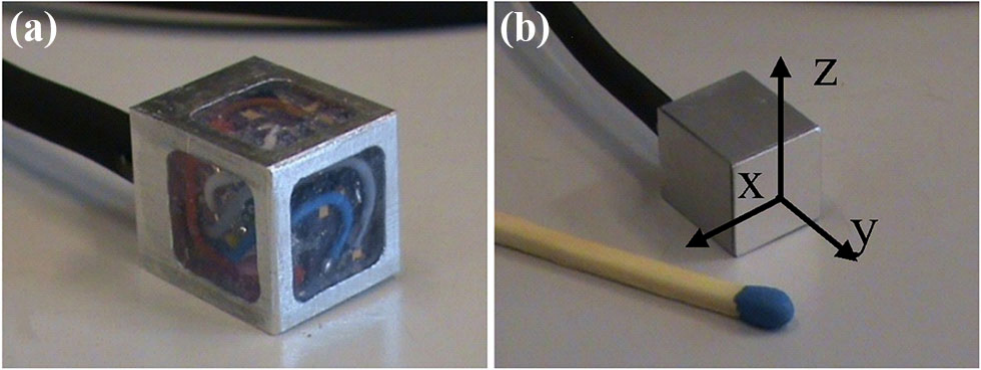
(**a**) Large-scale sensors (3D accelerometers (two 2D sensors) embedded in a cube as the first prototype. (**b**) The cube was sealed and glued on the tibial implant to measure the propagated vibration to the tibial part.

To measure the propagated vibration to the tibial implant, we sealed two dual-axis accelerometers (ADXL203) perpendicularly inside a metallic cubic case (11 × 11 × 13 mm^3^), to obtain tri-axial acceleration measurement with desired range and resolution (Fig. 2). The perpendicularity of sensors axes was tested prior to the experiments. The gain and offset of accelerometers in the sensor cube were obtained using Ferarris calibration.^14^ This cube is a large scale demonstrator of the sensors to be integrated into the smart prosthesis in the future.

### Experimental Protocol

We used 14 fresh-frozen lower limb specimens. This work aims at testing the feasibility of knee implants loosening detection, while no prior data were available to perform a power analysis. Cadavers (8 women and 6 men) with no infection history were selected by the local Institute of Anatomy (CHUV, Switzerland) from a donor program (mean age 85 y/o, range 46–100 y/o). The lower limbs were separated from the body at the upper third level of the thigh. No information about the age, height and weight of cadavers were available due to local ethical regulations. Macroscopic evaluation of the specimens during dissection allowed us to rule out the presence of any excessive subcutaneous adipose tissue and major bone defect or disease and showed that two of them were over-weighted. However fat subcutaneous tissue covering the medial side of the proximal tibia was considered low (< 1 cm) in all the legs. Legs were kept frozen at − 8° Celsius and kept at room temperature the day before the experiment. A size-3 tibial component of F.I.R.S.T TKA (Symbios SA, Switzerland) was implanted and cemented in each leg by a senior orthopedic surgeon and no implant was bigger than the bone surface, suggesting a similar tibia cut surface area across the specimens. The surgical procedure was as follows: midline skin incision, centered on the middle of the patella, followed by medial parapatellar arthrotomy, removal of the menisci and both cruciate ligaments, and tibial preparation using an extramedullary guide for a posterior-stabilized knee implant. The tibial cut was then cleaned using pumping water and drying, followed by fixation of the tibial implant using 40 grams of Palacos R bone cement (Palacos R, Zimmer, USA). We allowed the cement become solid for 15 min. Cemented implantation was chosen due to the exclusive experience in the medical institution, possibility of a closer-to-reality simulation of secure and loose implants for cadaver legs since the integration of cementless TKA in cadaver specimen is impossible.

**Figure 3:**
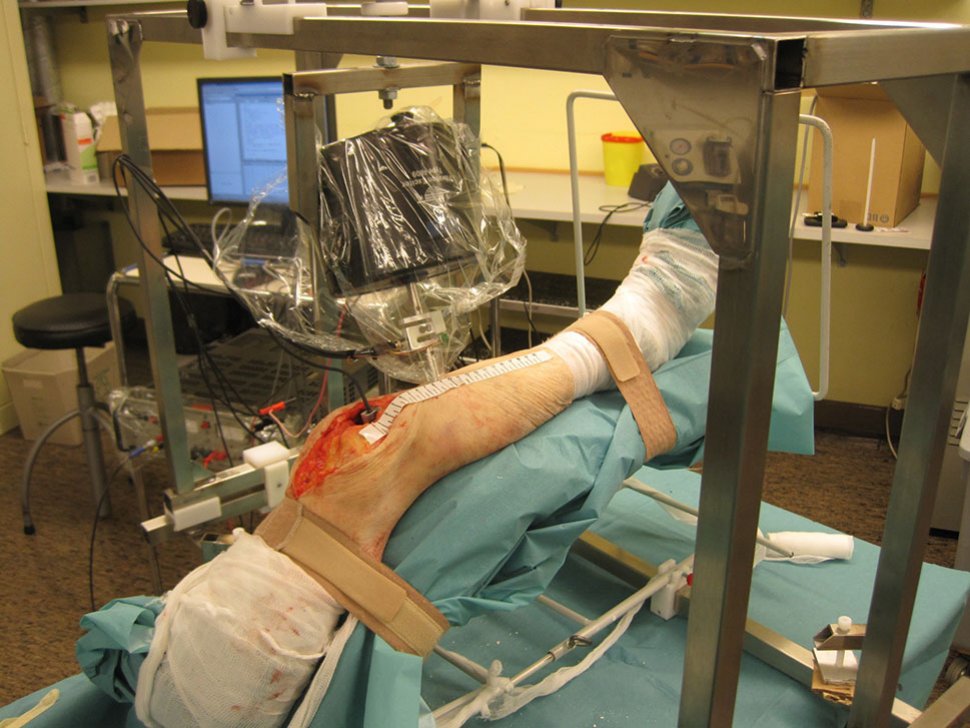
Vibrator and sensor cube location during the experiment.

The sensor cube was glued (Loctite 420, Loctite Corp., USA) on the tibial component. Each leg was then firmly fixed to the bed with two elastic bands positioned at the thigh root and directly above malleoli. The bed was fixed in the adjustable apparatus. The vibrator was then manipulated to vibrate 10 cm below the lower tip of the patella with a perpendicular impact angle with respect to the tibial crest (Figs. 1, 3). This impact point was chosen to facilitate the measurement repeatability. The vertical position of the vibrator was adjusted prior to the stimulation, to maintain a 5 N force on the contact. The vibration was applied at very low amplitude (always smaller than 0.39 mm).

Each experiment lasted 4 min during which the input vibration, contact force, and propagated 3D vibrations to the tibial implant were measured. Thereafter the surgeon systematically removed the cement layer from the bone-prosthesis interface with a 2-centimeter wide bone chisel. The removal was performed under the entire surface of the tibial tray and around the stem to be closest to clinical findings that demonstrated that tibial tray loosening happened all around the implant.^17^ The implant was then manually separated from the bone that is currently the criterion for confirming the implant loosening during prosthesis surgical revisions.^27^ After replacing the implant by press fit, and repositioning and adjusting the vibrator to get the same impact angle and contact force, we repeated all measurements. To investigate the repeatability, each measurement was performed at least twice after the vibrator was moved away from the leg and replaced.

The experimental protocol was approved by the research ethics committee of the faculty of medicine at the University of Lausanne.

### Signal Processing and Detection Methods

Input–output coherence was used to detect implant loosing (2.3.1). While for the sake of comparison, other frequency domain detection techniques (described in 2.3.2) were implemented.

#### Input–Output Coherence

Coherence is a bounded measure of linear association between two signals.^36^ In our work, it was used to indicate to what extent the output vibrations could be predicted by a linear function of input vibrations. As such, Coherence (*C*_*sx*_) between the stimulation (*s*) and axial output vibration (*x*) is defined as:

1 $$C_{sx} (f) = \frac{{\left| {S_{sx} } \right|^{2} }}{{S_{ss} S_{xx} }}$$

where *S*_*sx*_, *S*_*ss*_ and *S*_*xx*_ are the estimated cross-spectral density, and estimated auto-spectral densities of *s* and *x*, respectively. In order to compare the coherence of different trials, the Pearson correlation coefficient between the coherences (either between repeated measurements of secure implants or between secure and loose implants) was calculated and compared to a threshold to detect any potential loosening. The effect of different thresholds was demonstrated on a Receiver Operating Characteristic (ROC) curve.

#### Power Spectrum Density Estimation

Power spectra of the axially propagated vibrations to the tibial plate were computed. Considering the peaks of these spectra as the landmarks of energy concentration, we extracted a pattern. The power spectrum (*S*(*f*)) of a signal is the Fourier transform of its autocorrelation function (*R*_*xx*_):

2 $$S\left( f \right) = \int_{ - \infty }^{\infty } {R_{xx} (\tau )} e^{ - j\omega \tau } {\text{d}}\tau.$$

We applied Welch power spectrum estimation^47^ with a Blackman windowing to reduce amplitude errors using MATLAB (Mathworks, USA). Then four features were extracted from the power spectra: (i) number of peaks in 750–900 Hz frequency band, (ii) peak shift at 700–1200 Hz band, (iii) peak shift at 1200–2200 Hz band, and (iv) peak flattening at 500–1500 Hz band.

First, peaks were extracted automatically as the points with larger amplitude than the two left and two right neighbors, representing a neighborhood window of 15.625 Hz, in the Welch spectra. For detecting a peak shift, only the peaks with the shifted frequency of more than a threshold of 70 Hz were considered. For detection of peak flattening, first, a Gaussian was fitted to a window around each peak. Then, the peak width was calculated as the distance between the mid-amplitude points of the Gaussian, also known as the full width at half maximum. Assuming that each peak can fit a Gaussian function with a standard deviation of *σ*, we computed its width using Eq. 3. A threshold of 100 Hz was used to detect if a peak was flattened, in other words, if its width increased more than 100 Hz.

3 $${\text{Peak}}_{\text{width}} = 2\sigma \sqrt {2{ \ln }\left( 2 \right)}.$$

#### Validation

Since two or three repetitions of trials were performed for each leg, for secure and loosened case, subsamples of measurements were used to evaluate sensitivity, specificity, and accuracy of different methods. Each subsample contained a secure and a loosened measurement trial for each specimen. Subsamples were chosen such that every combination of measurements per specimen included in data analysis. Then the expected value and standard deviation of each performance metric were estimated for each specimen and then across the 14 legs.

In order to investigate the robustness of output acceleration power spectra, the intra-class correlation (ICC) was computed for each specimen (leg) on the spectra resulted from the repeated measurements at each condition (secure or loosened). The mean and standard deviation of ICCs for each condition were then calculated over the 14 legs. The frequency at which the power spectra peaks appear were estimated and compared statistically between the secure and loosened cases using Wilcoxon rank sum test with a significance level of 0.05. The choice of statistical test was made due to the low number of samples and the expected not-Normally distributed frequencies of the peaks.

A McNemar test with Bonferroni correction was used to compare the proportions of successful detection. Besides, to statistically compare the sensitivity, specificity and accuracy of different detection techniques, a Friedman test was performed to investigate the group-level statistical differences. Then, Wilcoxon signed rank test with Bonferroni correction was performed between each pair of techniques.

## Results

### Input–Output Coherence

**Figure 4:**
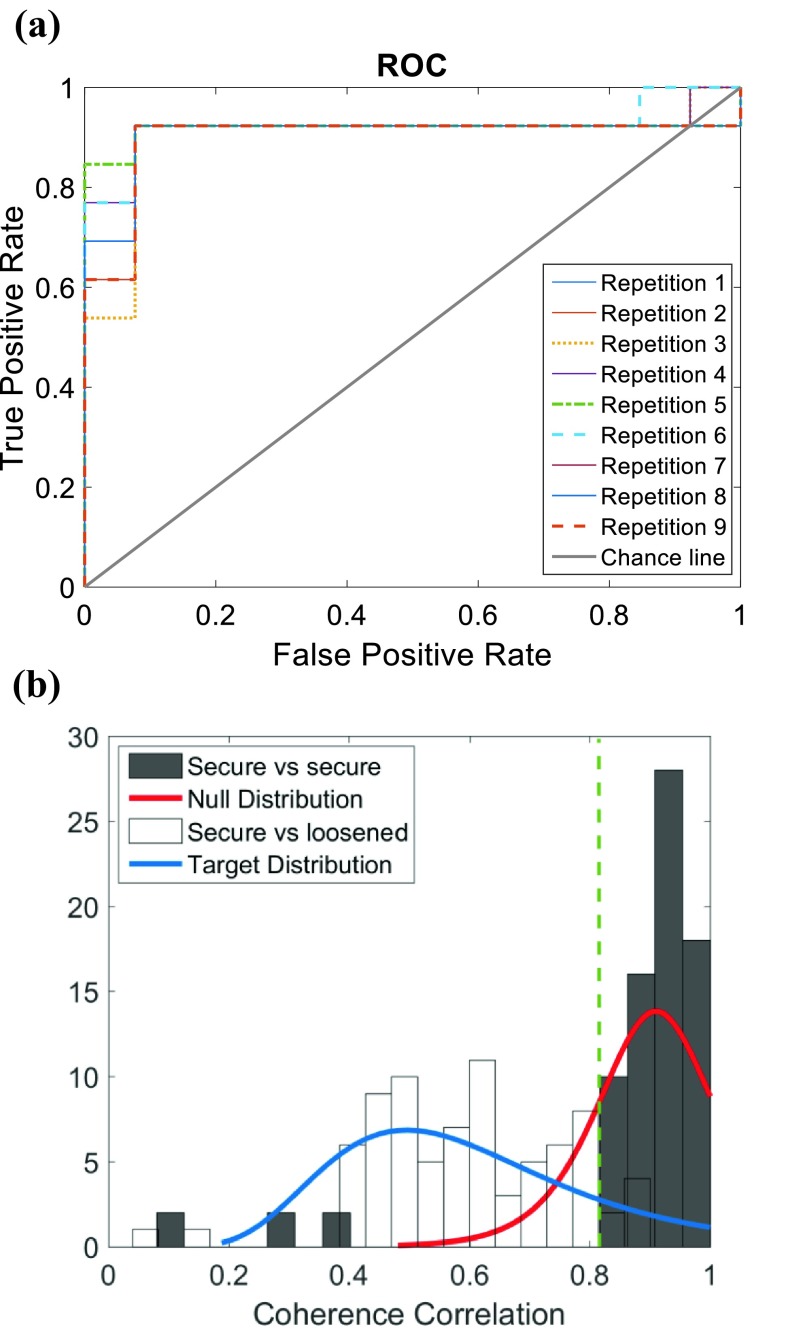
(**a**) ROC curve for different samples of repetitions of experiments for the 14 legs, plotted against the chance line for the binary decision. As they were three repetitions of each measurement in secured and loosened conditions, the ROC curves of 9 possible combinations (repetitions) were plotted; (**b**) the coherence correlation distribution for secure and loose implants. Dark bins showed the secure implants coherence correlation of repeated measurements, light bins show the dispersion of coherence correlation between secure and loosened case. The green dashed line shows the selected threshold. Also, two probability density functions are fitted to the secure (Null) and loosened implants (target).

Figure 4a demonstrates the True Positive Rate against the False Positive Rates for different applied thresholds on the correlation between the obtained coherences from different trials. The distribution of correlation between the coherences for the secure implants and loose implants are demonstrated in Fig. 4b.

The correlation coefficient between the coherences computed for the repeated measurements of secure implants was 0.87 ± 0.19 (mean ± SD over the 14 legs), while the correlation between the coherences computed for the corresponding secure (baseline) and loosened implants decreased to 0.60 ± 0.15. The optimal threshold for distinguishing loosened implants from secure ones was selected at 0.82 based on Fig. 4 leading to the highest expected accuracy, sensitivity, and specificity of 92.26, 91.67 and 92.86% respectively (Table 1).

**Table 1:** Performance analysis of implant loosening detection built upon, input–output coherence correlation and different output spectrum features.

	Coherence correlation (%)	New peak in (750–900 Hz) (%)	Peak shift in (700–1200 Hz) (%)	Peak shift in (1200–2200 Hz) (%)	Peak flattening (%)
Sensitivity	91.67 ± 0.03	77.2 ± 6.8	53.1 ± 13.7	66.2 ± 9.0	51.0 ± 4.6
Specificity	92.86 ± 0.00	78.9 ± 3.9	74.3 ± 10.5	43.9 ± 5.8	84.6 ± 10.2
Accuracy	92.26 ± 0.01	78.0 ± 3.5	63.0 ± 5.8	57.2 ± 8.1	67.3 ± 6.0

The mean and standard deviation of each metric were obtained over repeated measurements over 14 legs

In addition, a correlation coefficient of 0.92 ± 0.07 was obtained between the coherences computed for the repeated measurements of the loosened implants.

### Power Spectrum Density Estimation

The power spectrum of the measured output vibration showed variable repeatability across the legs on tridimensional axes. Since the most repeatable pattern was obtained in the longitudinal axis, the vibration analysis was performed exclusively on this axis.

Power spectra for each leg were estimated in two cases: after implant cementation, considered as a baseline (well-fixed), and after total implant loosening.

**Figure 5:**
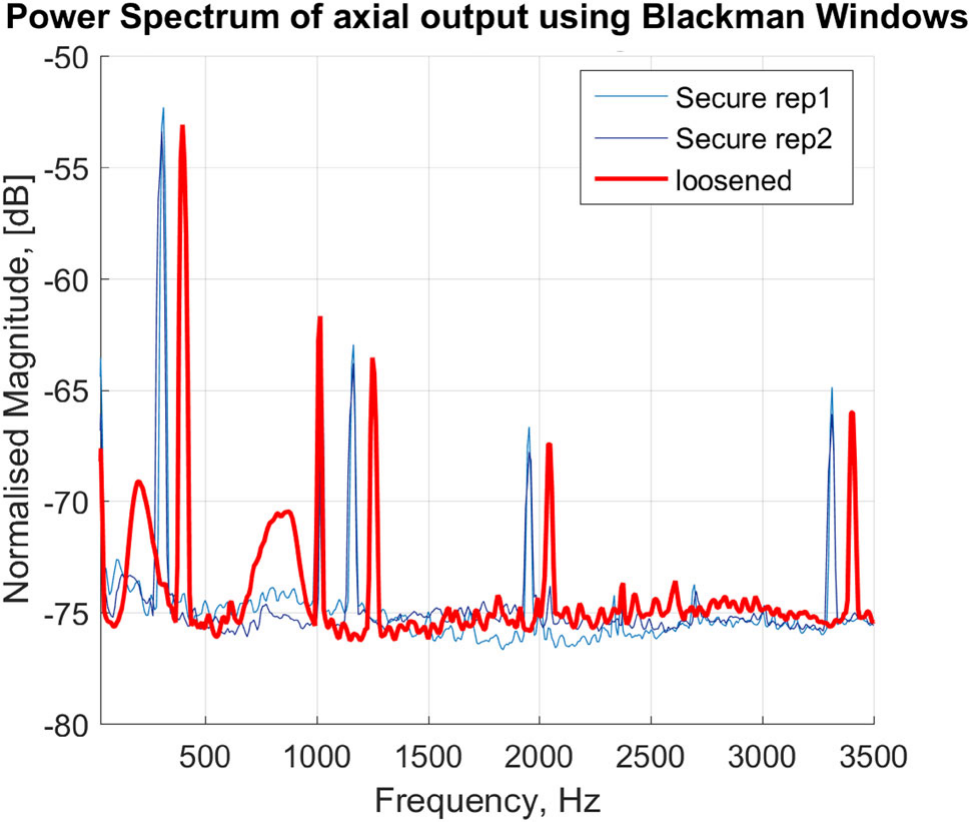
Welch power spectra of axially propagated acceleration (vibration) to the tibial implant for a representative; secure implant (thin lines in blue) and loose implant (thick line in red).

Tibial implant loosening was characterized by the appearance of a new peak between 750 and 900 Hz (Fig. 5). This peak appeared in 11 out of 14 total implant loosening cases. In eight legs, the new peak appeared between the first and the second baseline peaks. In two cases the new peak appeared between second and third and in one case between third and fourth peaks of the baseline spectrum. Repeated measurement of the vibration propagation on each condition was analyzed and summarized in Table 1, i.e., sensitivity, specificity and accuracy of the method in detection of secure or loosened implants. It must be noted that the new peak did not always appear in the repeated measurements which resulted in an expected sensitivity and specificity of 78.9 and 77.2% respectively.

Peak shifts were examined as an indicator of loosening. While, in general, a shift to the left side of the spectrum can represent the loosening, in some cases, a peak split can instead appear. The peak split might be observed as the shifting of peaks to the both left and right side of the frequency band.

**Figure 6:**
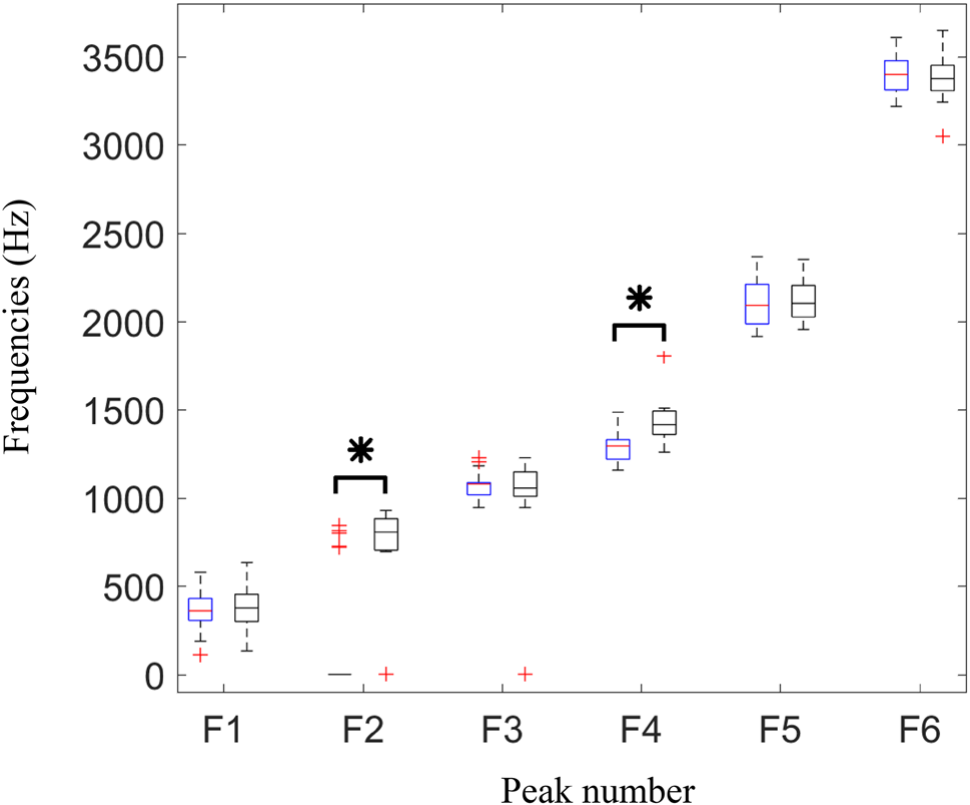
Frequencies of the six dominant peaks detected in power spectra in secure (left boxes in blue) and loosened (right boxes in black) tibial implants. Results are depicted as boxplot for 14 legs. Asterisk (*) indicates significant difference between the frequencies detected for secure and loosen cases (significance level of 0.05).

The frequencies of the detected peaks are shown in Fig. 6. As demonstrated in the boxplots, Normal distribution cannot be assumed for the frequencies, therefore a Wilcoxon rank sum test performed in each peak clusters between the secure and loose implant samples (Fig. 6). The test showed two significant differences in the frequencies of two peak groups, i.e., second peaks (mostly around 750–1000 Hz) and fourth peaks (mostly in the band of 1200–1800 Hz), with *p* values < 0.0001.

The appearance of multiple peaks in a close frequency band can possibly result in flattening of a spectrum peak computed *via* Welch method. Therefore, the peak flattening results (Table 1) showed high specificity.

In test–retest repeatability analysis of the power spectra, no change in the number of peaks was observed in the measurements of 11 legs, while in two legs a peak in the power spectrum sometimes was not observed in repeated measurements of secure implant. In another leg, one of the peaks in the power spectrum sometimes was not observed both in repeated measurements of secure and loosened implant. ICCs of obtained spectra for sampled repeated measurements were 0.86 ± 0.08 and 0.97 ± 0.02 (mean ± SD over the 14 legs) for secure and loosened cases, respectively.

**Figure 7:**
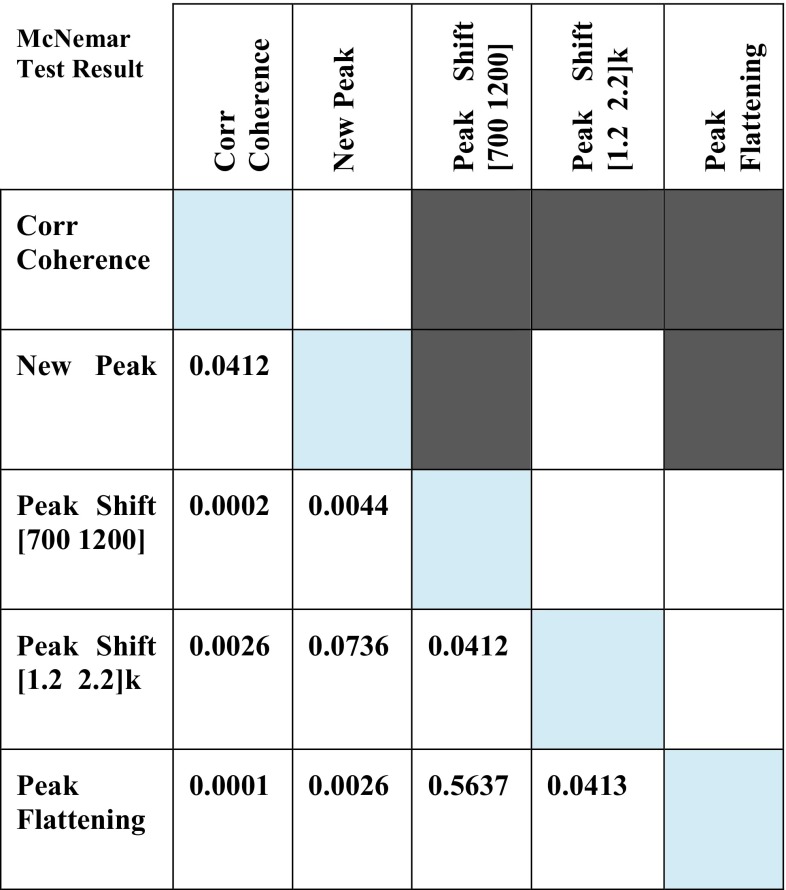
Statistical comparison of proportions of successful implant loosening detections among different methods, i.e., input–output coherence, new peak appearance in output spectrum, Peak shift in (700–1200 Hz), Peak shift in (12000–2200 Hz), and peak flattening using McNemar test with Bonferroni correction (*α* = 0.05/10). Each row and column in the colored table represent the corresponding detection method, and dark gray color indicates a significance difference. The number on the bottom half of the table are the obtained *p* values.

**Figure 8:**
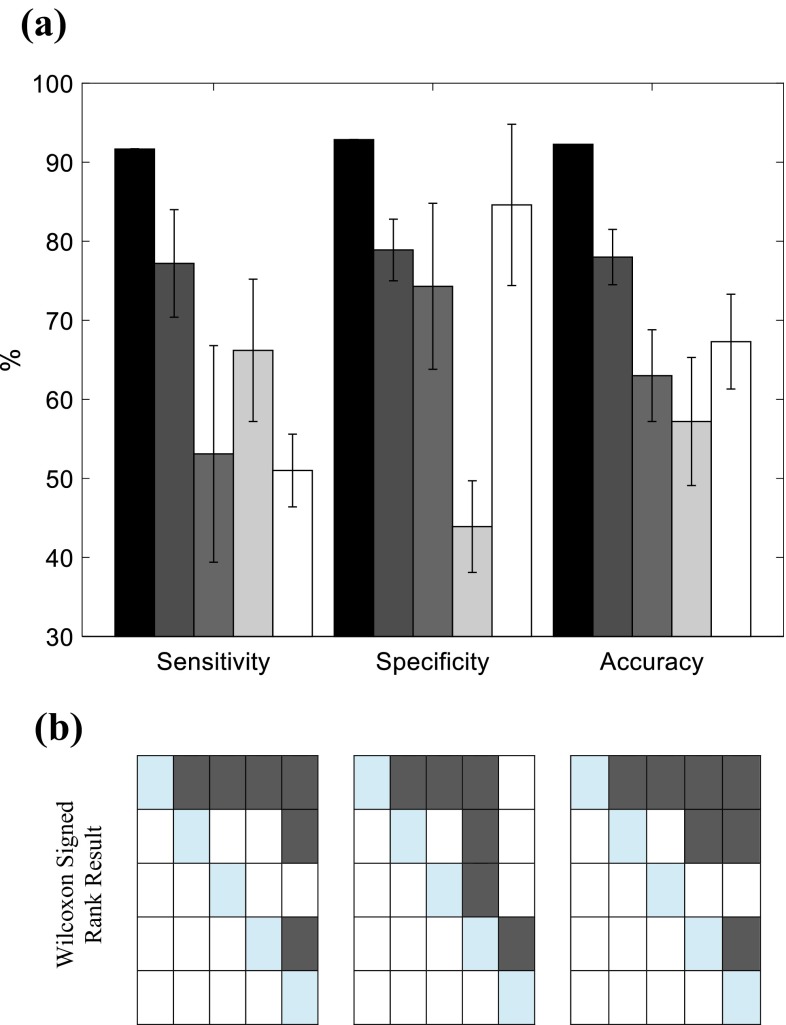
Statistical comparison on sensitivity, specificity and accuracy of different loosening detection methods. (**a**) Barplots show the mean ± SD of sensitivity, specificity and accuracy of input–output coherence (black), new peak appearance in output spectrum (dark gray), Peak shift in (700–1200 Hz) (gray), Peak shift in (12,000–2200 Hz) (light gray), peak flattening (white). (**b**) Result of Wilcoxon signed rank test between each pair of techniques with Bonferroni correction where dark gray in each table showed the significant difference: sensitivity (left), specificity (middle), and accuracy (right) significant differences are indicated across the input–output coherence, new peak appearance in output spectrum, Peak shift in (700–1200 Hz), Peak shift in (12,000–2200 Hz), and peak flattening, represented by rows/columns 1–5 respectively.

The result of McNemar test on the proportions of successful loosening detection is depicted in Fig. 7. The correlation coefficient between the computed input–output coherences of secured and loosened implants showed significantly better detection results than peak shift and peak flattening in propagated vibration spectrum. Moreover, the appearance of new peak showed significantly better detection results than peak shift in 700–1200 Hz and peak flattening. Friedman test on the sensitivity, specificity and accuracy of the implant loosening detection methods yields *p* < 0.000004. The Wilcoxon signed rank tests between each pair of loosening detection techniques (Fig. 8), with Bonferroni correction, revealed significant differences in sensitivity and accuracy between the coherence based detection and output vibration based techniques. The coherence based technique showed significantly higher specificity than loosening detection based on a new peak or peak shift in output vibration spectrum.

## Discussion and Conclusion

In this work, we presented the proof of concept of a vibration analysis based system to detect the tibial implant loosening in cemented knee prostheses for post-operative follow-up. To the best of our knowledge, this is the first vibration analysis study for tibial components loosening detection in knee prostheses. Our system works based on transcutaneous stimulation of the crest of the tibia and measuring the propagated vibration using a 3D accelerometer unit firmly attached to the tibial implant. Input–output vibration coherence was used to detect the implant loosening and benchmarked against the output vibration frequency domain analysis techniques used for hip implants in the previous studies. 14 lower limb specimens were tested, a much larger sample size than existing studies on THA, to evaluate the performance of the detection techniques. The proposed coherence-based technique, with 91.67% sensitivity and 92.86% specificity outperformed the other methods which exclusively relied on the output vibration propagated to the implant.

Among the tested output vibration frequency domain features, the appearance of a new peak and peak flattening demonstrated high sensitivity (77.2%) and specificity (84.6%). Obtained high repeatability of power spectra and input–output coherence was associated with our proposed instrumentation that allowed producing highly repeatable vibrations stimulation. This beside simplicity of adjusting and performing the test, i.e., 2 to 3 min to reorient the vibrator and 4 min to perform the experiment, suggested a great potential for translating the proposed system to loosening evaluations of instrumented knee implants in clinical practice.

The output power spectrum techniques relied on the analysis of the vibration measured by fixed accelerometer cube on the tibial implant, and the implicit assumption of identical stimulation for different loosening states. Close scrutiny on the peaks of the spectra revealed that after implant loosening, a new peak appeared in the 750–900 Hz band at 11 out of 14 legs. The appearance of the new peak which is in accordance with several reported results in THA loosening detection,^16^,^35^,^46^ can be justified as a new mode in the tibia-cement-implant dynamics caused by the broken cements and micromotions. The peak shifts showed to be less sensitive to loosening with sensitivities less than 70%. It must be noted that a peak split occurred in several cases due to the implemented loosening which could be observed as the shifting of peaks to the both left and right side of the frequency band. This ambiguity could be contributed to the inferior performance of loosening detection based on the shift of peaks when comparing to loosening detection based on the appearance of a new peak. The peak flattening showed however to be a specific feature which can be used to reduce the false negatives. Coherence analysis, in contrast to the previous methods, is less sensitive to the variations of the input stimulation across the experiments. However, it relies on a linear system assumption which is not preserved particularly for the loosened implants.^24^,^37^ Nevertheless, significant difference was observed between the correlation of coherence before and after the implant loosening with the baseline coherence (coherence in the secure implant). The coherence-based method also obtained the highest accuracy 92.26% among the other methods, and can thus be recommended for implant loosening detection.

In general, the explored features showed encouraging outcome in the detection of totally loosened implants. Our approach to artificially creating the loosening was confirmed by the surgeons to be similar to the natural loosening observed for *in vivo* cases. Since our hypothesis was that the implant loosening changes the mechanical contact between the implant and bone, our approach to creating artificial loosening was a necessary step in the validation of our proposed method.

Notably, the correlation coefficients between the coherences calculated for the two repeated measurements of the secured implants and loosened implants were around 0.90. Although slightly larger in the latter case, this difference is not statistically significant (Wilcoxon rank sum test *p* value > 0.51). These high correlations indicate the repeatability of the measurements in both cases. At the same time, the correlation coefficient between the coherences computed for the corresponding secure and loosened implants was around 0.60. This low correlation shows a large difference between coherences measurements of secure and loosened implants. This considerable difference (0.90 vs. 0.60) indicates the introduced coherence correlation as a robust measure for loosening detection.

Considering the complex geometry and material properties of the tibia and tibial implant, and the boundary conditions at their cemented interface, we did not expect to have all the peaks in similar frequency bands to THA studies and have vibration propagation exclusively in the excitation plane. Instead, we expected to observe a 3D micromovement of the implant as the result of vibration propagation throughout the bone. Despite designing the cube to provide 3D acceleration measurement, the power spectrum of the measured acceleration showed lower repeatability across the specimens in the traverse axes than the longitudinal axis. Since the most repeatable patterns were obtained in the longitudinal axis, the vibration analysis was performed exclusively on this axis. The reason for lower repeatability in transverse axes might be two folds. First, the tibia could have slightly rotated around its longitudinal axis on the implantation bed that could cause crosstalk in the two transverse components of the accelerometer readout. Because of such specimen placement error, we expect to have higher inter-specimen variability in each of these two transverse components. Second, the vibration propagated longitudinally along tibia and thus its amplitude measured on the implant was larger in the longitudinal direction leading to larger signal to noise ratio and thus higher inter-specimen repeatability.

Despite exclusive vibration analysis on the longitudinal axis, the 3D acceleration measurement is beneficial for controlling and repeating vibration tests, particularly for the leg placement. When the leg is in a static position, the accelerometers (off-axial channels) could be used as inclinometers, based on the projection of the gravitational force, to correct the orientation of the leg. The 3D accelerometer could also be be fused with other external sensors for kinematic measurements.^3^ Although this study was a proof of concept and focused on the feasibility of the loosening detection method, the other aspects of the smart implant design, namely remote powering and wireless communication units,^4^,^6^ supplementary electronics and packaging have been addressed in our other studies.^15^,^45^ The discussed sensor cube, fixed on the tibial tray, needs to be miniaturized and embedded in the final smart prosthesis together with the supplementary electronics.

In the current study, only one size of tibial cemented implant was used to provide a fair comparison in a rather small sample size. Experiments with the other types and sizes would be necessary for future. Also it is worthy to note that no femoral component was implanted in this study due to isolating the effect of loosening at the tibia-prosthesis interface. We expect that the contact of tibial and femoral parts in a prosthetic knee would minimally alter the results since this contact is much looser than the loosened-cemented contact between the tibia and tibial implant. Nevertheless, this effect should be investigated in the future.

Bone density, fat content and leg size could influence the output power spectra and input–output coherences across different specimens. While the difference in bone density and leg size can result in peak shift in the frequency bands of interest for implant loosening detection, the fat content is expected to affect mostly the low-frequency component of both output power spectrum and input–output coherence. In the present study, since each leg was compared to itself (its baseline results) no significant change in the detection performance is expected due to the fat density, leg size and bone density. Nevertheless, the effects of these features on the frequency response of the bone-implant should be investigated in the future studies.

Also further studies can be performed to examine the possibility of localizing the defect in the bone-cement-implant interface by employing the result of studies in structural damage detection and localization.^10^,^18^,^41^ The comparison of our results with current radiological techniques was not relevant due to the low accuracy of X-ray for implant loosening detection.^43^ Alternatively, the techniques such as bone scintigraphy, which has higher accuracy, cannot be performed on cadaver specimens.

This study focused on the cemented tibial component loosening due to the possibility of simulating it in *ex vivo* legs and the exclusive experience with cemented TKA in our institution. The proposed system can also be used for cementless implants in future.

In this study, we proposed sensitive and objective loosening detection techniques that do not depend on the physicians’ subjective views. These techniques could be easily translated into clinical practice where the results of vibration analysis on each leg would be compared to itself. The inter-subject differences in bone quality, soft tissue density and volume, and the implant type would minimally affect the implant loosening detection. This was reflected in high specificity of our proposed implant loosening detection techniques.
